# Head posture impacts mammalian hyoid position and suprahyoid muscle length: implication for swallowing biomechanics

**DOI:** 10.1098/rstb.2022.0552

**Published:** 2023-12-04

**Authors:** Peishu Li, Callum F. Ross, Zhe-Xi Luo, Nicholas J. Gidmark

**Affiliations:** ^1^ Department of Organismal Biology and Anatomy, University of Chicago, Chicago IL, 60637, USA; ^2^ Department of Biology, Knox College, Galesburg IL, 61401, USA

**Keywords:** biomechanics, muscle physiology, mammal evolution, swallowing, *Didelphis virginiana*

## Abstract

Instantaneous head posture (IHP) can extensively alter resting hyoid position in humans, yet postural effects on resting hyoid position remain poorly documented among mammals in general. Clarifying this relationship is essential for evaluating interspecific variation in hyoid posture across evolution, and understanding its implications for hyolingual soft tissue function and swallowing motor control. Using *Didelphis virginiana* as a model, we conducted static manipulation experiments to show that head flexion shifts hyoid position rostrally relative to the cranium across different gapes. IHP-induced shifts in hyoid position along the anteroposterior axis are comparable to *in vivo* hyoid protraction distance during swallowing. IHP also has opposite effects on passive genio- and stylohyoid muscle lengths. High-speed biplanar videoradiography suggests *Didelphis* consistently swallows at neutral to flexed posture, with stereotyped hyoid kinematics across different head postures. IHP change can affect suprahyoid muscle force production by shifting their positions on the length-tension curve, and redirecting lines of action and the resultant force from supra- and infrahyoid muscles. We hypothesize that demands on muscle performance may constrain the range of swallowing head postures in mammals.

This article is part of the theme issue ‘Food processing and nutritional assimilation in animals’.

## 1. Introduction

The mammalian hyoid is often described as a ‘floating’ bone [1]. In many lineages the hyoid is connected to skull and postcranium by soft tissues only [2,3]. Resting hyoid position in humans and other anthropoids has long been of interest for investigating the anatomical basis of speech [4–7], and recent discoveries have uncovered new diversity of resting hyoid position across mammals more broadly [3,8–10]. Interspecific variation in resting hyoid position has been postulated to reflect changes in craniomandibular and hyoid morphology [3,6,11] and carry functional implications for vocalization and swallowing biomechanics [2,8,9,12]. Nonetheless, testing these hypotheses on the form-function relationship of resting hyoid position remains challenging in a comparative framework.

Within individuals, resting hyoid position also varies with instantaneous head posture (IHP) [13–15], defined as any head posture within the full posture space of an awake animal. The coupling relationship between hyoid position and IHP has been well established in humans [13,16,17] yet poorly documented in other mammals [14,15]. Addressing this knowledge gap is critical for evaluating hypotheses of evolutionary disparity in resting hyoid position. It also offers insights into the biomechanical relationships between resting hyoid position and hyolingual function within individuals before such relationships can be tested in a broader evolutionary context.

It remains unclear how IHP's effect on resting hyoid position relates to swallowing performance, which requires precise control of hyoid position over a rapid timescale. With little skeletal constraint, hyoid position at any given time depends on the geometry and activity patterns of supra- and infrahyoid muscles [1], and interaction with surrounding structures such as the mandible. During swallowing, supra- and infrahyoid muscles undergo complex changes in gearing [12], contractile pattern [10,18] and geometry [10,12,18] to drive hyoid movement for airway protection [19], upper oesophageal sphincter opening [20], and—in some primates at least—tongue base retraction [21]. Any change in resting hyoid position may affect how the hyoid muscles function dynamically. In support of this hypothesis, computational modelling of human, macaque and rabbit hyolingual systems suggest that hyoid range of motion varies with different hyoid starting positions, muscle lengths and force vector orientations [12,22,23]. However, so far the effect of IHP on hyoid muscle geometry and *in vivo* function is poorly understood [24].

Coupling between IHP and static hyoid position suggests animals should be capable of using head posture to modulate hyoid position to achieve optimal swallowing performance. Head–neck or head–trunk interactions are often necessary for successful food transport in vertebrates [25–28]. Human dysphagia patients are sometimes encouraged to adopt a specific head posture during swallowing [29,30], yet swallowing head posture has not been systematically documented across mammals in general [31]. In theory, individuals may adopt certain swallowing head postures for several, non-mutually exclusive reasons, including priming hyolingual muscle geometry for optimal control of hyoid position [24], and/or manipulating spatial dimensions of the pharynx for safer bolus transit [29,32–34]. Testing these hypotheses requires integrating IHP and resting hyoid position measurements with observations in hyolingual muscle geometry, hyoid kinematics and overall swallowing performance [24,35].

Here we address three questions using the Virginia opossum (*Didelphis virginiana*) as a model: (i) how does IHP impact hyoid position and suprahyoid muscle length across different gapes? (ii) do static hyoid position and suprahyoid muscle lengths differ significantly between different head postures across gape? and (iii) given potential interaction between IHP, hyoid position and muscle geometry, what head posture(s) would individuals adopt during *in vivo* swallowing? To address questions (i) and (ii) we conducted static manipulation experiments in anaesthetized individuals to quantify hyoid position across different head postures and gapes. For each posture-gape combination, we also measured the lengths of two suprahyoid muscles important for hyoid movement: geniohyoid and stylohyoid [36,37]. We hypothesized that head extension would significantly alter hyoid-cranium position [13], lengthen geniohyoid [35] but shorten stylohyoid. To address question (iii), we used the XROMM workflow [38] to quantify *in vivo* swallowing head posture and hyoid kinematics. We hypothesized that an animal would only use a subset of its total head posture range during swallowing; within this range, hyoid kinematics should differ in excursion magnitude and/or timings between different swallowing head postures.

## 2. Material and methods

### (a) Subjects

Eight adult *D. virginiana* (three males and five females) were locally captured in Chicago, IL (IDNR permit number W22.6676). Six individuals (three males, three females) were statically manipulated to measure hyoid position change across different posture-gape combinations (see §2b, below). The other two female individuals were used for *in vivo* kinematic data collection (see §2f, below). All experimental procedures were approved by the University of Chicago Animal Care and Use Committee.

### (b) Computerized tomography scanning and data collection on static hyoid position

**Figure 1.**
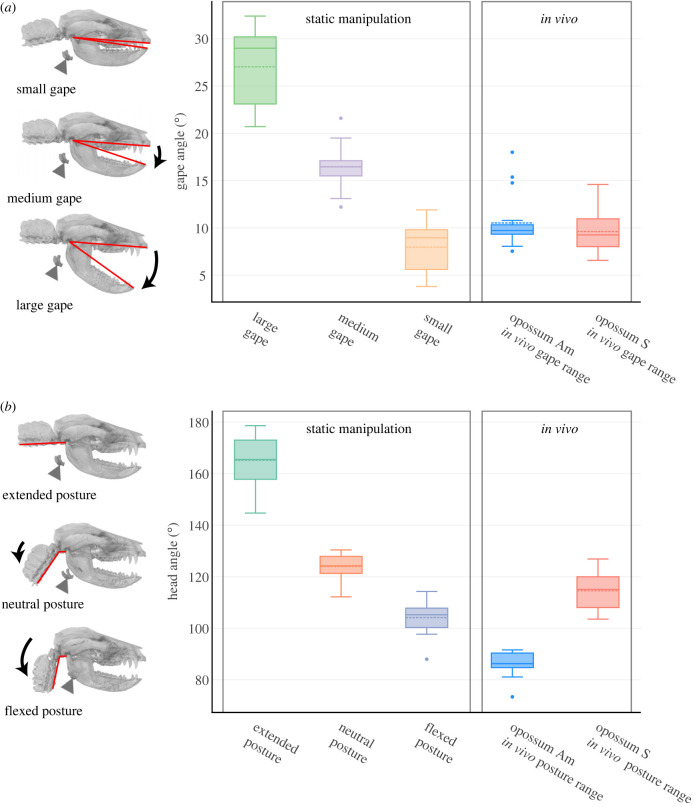
*In vivo* changes in *Didelphis* head posture and gape during swallowing occupy a subset of the ranges in static manipulation experiments. (*a*) shows gape angle range in each static gape group and the observed range of gape movement from experimental individuals during swallowing. (*b*) shows head flexion angle angle in each static head posture group in comparison with the average within-cycle head flexion angle during swallowing. Dashed line in each boxplot denotes mean values while solid line denotes median. Three-dimensional visualization shows representative head and gape posture within each static manipulation group. Red lines indicate the measurement scheme of gape angle and head flexion angle, respectively. Triangle indicates hyoid position.

Six individuals were anesthetized via intramuscular injection of Alfaxolone (7–10 mg kg^−1^) and Midazolam (0.3–0.5 mg kg^−1^). Using a Vimago veterinary computerized tomography (CT) scanner, we scanned each individual at three head postures (extended, neutral and flexed) and three gape angles (small, medium and large), for a total of nine scans of distinct head-gape posture combinations per individual (*n* = 54 scans for all six individuals) (electronic supplementary material, figure S1A). Scan parameters are given in the electronic supplementary material, table S1. For extended head posture (144.7–178.6°; figure 1*a*), we placed the animal prone with head extended in the midline. For flexed head posture (88–114.3°), we placed the animal on its right side, tucked the head underneath the forelimbs and secured it with tape. For the neutral posture (112.2–130.4°), we extended the head out of the flexed posture and placed it roughly halfway between the extended and the flexed posture. At each head posture, small gape (3.8–11.9°) was achieved with upper and lower teeth in occlusion. We placed a plastic mouth prop either horizontally or vertically between upper and lower canines to achieve medium (12.2–21.6°) or large (20.7–32.4°) gape, respectively. We always started scanning at extended head posture and small gape, followed by large and medium gape at the same head posture, before repeating the sequence at the other two head postures. Individual size as measured by hemimandible length ranged from 88.7 to 107.6 mm.

During anaesthesia, the hyoids are not subject to any soft-tissue actuation. Several animal models have shown minimal hyoid muscle activity and length change during both anaesthesia and awake spontaneous breathing [35,39–43]. Therefore, the static hyoid position measured at a given posture-gape combination is a reasonable proxy for the true resting hyoid position in awake, spontaneously breathing animals with the same posture-gape combination.

CT scans were imported into Avizo v. 9.5.0 (Thermo Fisher Scientific, MA), and three-dimensional surface meshes of cranium, hemimandibles, hyoid and cervical vertebrae were segmented via automated thresholding and manual tracing. The meshes were cleaned, smoothed, and exported in .obj format, then further scaled by 1/1000 in Meshlab (v2020.03, [44]) to convert mesh unit to millimetres. We measured gape as the angle between two lines connecting the upper and lower incisors to the temporomandibular joint in lateral view (figure 1*a*; electronic supplementary material, figure S1), and we measured head posture as the angle (head flexion angle hereafter) between a line running along the external midline of the basioccipital to the atlantooccipital joint, and another line starting at the atlantooccipital joint and running along the ventral midline of C1-C3 (figure 1*b*; electronic supplementary material, figure S1).

**Figure 2.**
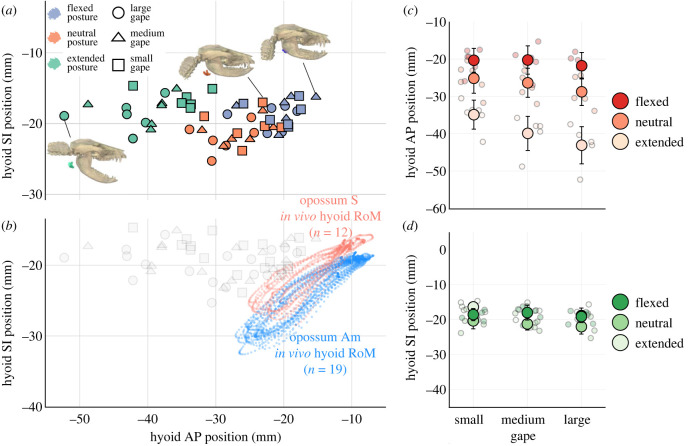
Head flexion shifts static hyoid position rostrally relative to the cranium in *Didelphis*. Rostral shift in hyoid position is comparable with *in vivo* hyoid protraction magnitude during swallowing. (*a*) shows static hyoid position across posture-gape combinations in the horizontal (anteroposterior, AP) and vertical (superoinferior, SI) axes. Three-dimensional visualizations represent extended posture with large gape (left), neutral posture with small gape (middle), and flexed posture with medium gape (right). (*b*) shows *in vivo* hyoid range of motion (RoM) along the AP and SI axes during swallowing cycles, plotted alongside static measurements at different posture-gape combinations in grey. (*c,d*) show mean hyoid position at each posture-gape combination overlaid above individual measurements in each anatomical axes. Error bars are standard deviations.

Cleaned and scaled meshes were imported into Autodesk Maya (v2022.3). For a given individual, we first aligned the cranium (with the hemimandibles, hyoid and vertebral column parented) to the same position and orientation using the snap align function, followed by manual adjustment. We created an anatomical coordinate system (ACS) at the posterior nasal spine (PNS) and parented it to the cranium, with positive *x*-axis pointing rostrally parallel to the upper tooth row, positive *y*-axis pointing superiorly perpendicular to the hard palate, and positive *z*-axis pointing laterally towards the right (figure 1*c,d*). We created another ACS parented to the hyoid with the same orientation. Using key frame animation, we quantified hyoid *x*, *y* and *z* coordinates in the cranial coordinate system through different posture-gape combinations, with the first frame starting at extended posture + small gape. Changes in hyoid *x*, *y* and *z* position represent changes in relative hyoid position along anteroposterior (AP), superoinferior (SI) and mediolateral (ML) directions, respectively (figure 2).

### (c) Theoretical modelling of hyoid position change across instantaneous head posture change

Previous studies have attempted to correct for IHP effects on hyoid position by mathematically adjusting head and hyoid positions assuming perfect coupling between the cranium and hyoid [15]. Should this assumption be true, for every degree of head flexion/extension, the hyoid should move accordingly so that its position relative to the cranium is always constant. Here we test this assumption by comparing measurements of static hyoid positions to expected hyoid positions at different IHPs based on simple mathematical modelling. We calculated changes in expected hyoid position (or lack thereof) with IHP change under two different conditions. The first condition assumed the hyoid is perfectly coupled to the cranium across head extension/flexion (similar to the assumptions of [15]). Hyoid *x*, *y* and *z* coordinates relative to the cranium should thus be constant across head posture and gape change. The other condition assumed the hyoid is perfectly coupled to the vertebral column and *decoupled* from the cranium. Hyoid *x* and *y* coordinates relative to the cranium should thus become more negative with increasing head extension, following a sinusoid function. *z* coordinates should remain constant. Calculations for both conditions were made for each gape group (More details in the electronic supplementary material).

### (d) diceCT and data collection on suprahyoid muscle length

In addition to the anesthetized individuals scanned above, we micro CT (*μ*CT) scanned a *D. virginiana* specimen from Chicago, IL using a GE phoenix v|tome|x scanner. The specimen was then submerged in 2% Lugol's iodine (I_2_KI) solution for six months, and *μ*CT scanned again upon staining completion. We segmented out whole muscle-tendon units of geniohyoid bilaterally and of left stylohyoid and aligned the muscle model with the bone models to locate muscle attachment sites. Geniohyoid has a tendinous origin at the posteroinferior edge of the hemimandible just lateral to the mandibular symphysis and inserts broadly along the ventral edge of basi- and thyrohyal. A distinct median septum divides the left and right geniohyoid muscle bellies until the posterior one-third of the muscles near their hyoid attachment sites. Given the lack of a distinct left-right boundary in the posterior portion, we segmented out the whole geniohyoid muscle volume for an accurate anatomical representation. Stylohyoid originates from the paroccipital process on cranium and inserts on the distal tip of the thyrohyal. The attachment sites for both genio- and stylohyoid were confirmed via dissection and primary literature [36,45].

We located the same geniohyoid and stylohyoid attachment sites in the experimental subjects. Using the distance measurement in Avizo, we measured the three-dimensional Euclidean distances between attachment sites to estimate how the passive lengths of genio- and stylohyoid changed across head postures and gape. For geniohyoid, we measured the distance between the midpoint on the ventral edge of the basihyal and the posteroinferior edge of left hemimandible just lateral to the mandibular symphysis. For stylohyoid, we measured the distance between the distal tip of the thyrohyal and the paroccipital process on the left side (figure 4). Changes in muscle length across different posture-gape combinations were standardized to the measured length at medium gape and neutral head posture (*l_i_*).

### (e) Surgical procedures

The two remaining individuals were anesthetized with Alfaxolone (7–10 mg kg^−1^) and Midazolam (0.3–0.5 mg kg^−1^) via intramuscular injection and maintained under general anaesthesia via isoflurane. One millimetre tantalum markers were implanted in the crania and hemimandibles (four in the cranium, five in each hemimandible). One 1 mm marker was implanted in the ventral aspect of the axis (C2) and three 0.8 mm markers were implanted in the hyoid in a triangular constellation, with a central marker on the basihyal, flanked by two markers on the thyrohyals. All marker locations were confirmed using post-mortem CT scans.

### (f) *In vivo* data collection and processing

The animals recovered after surgery for at least one week before data collection. Over multiple days we recorded high-speed biplanar videoradiographic data at 200 Hz of both individuals feeding on wet cat food mixed with apple juice inside a plastic chamber. The food bowl was always presented at a consistent location on the box floor directly in front of the animal, and the animal was free to adjust its body position and head posture throughout the experiment. Marker movement was digitized in XMALab [46]. Thirty-one intercalated swallow cycles from both individuals (19 from opossum Am, 12 from opossum S) were digitized in total. A swallow cycle was defined as the time between the last minimum gape before swallow onset to the first minimum gape after swallow offset. Rigid body transformation matrices for the cranium, each hemimandible and the hyoid (rigid body error ≤ 0.01), as well as the three-dimensional *xyz* coordinates of the vertebral marker (reprojection error ≤ 0.2) were filtered with a built-in zero-lag, 30 Hz low-pass Butterworth filter 30 Hz and exported into Autodesk Maya (v2022.3).

Bone models of the cranium, hemimandibles and hyoid of each individual were created from CT scans. Bone models were animated in Maya using filtered transformation matrices. Hyoid translation throughout swallow cycles was calculated in a cranial coordinate system, with each individual's ACS set up in the same position and orientation as those used for the static manipulation experiments. Instantaneous gape was measured as the pitch angle at the left temporomandibular joint calculated between an ACS parented to the cranium and another ACS parented to the left hemimandible. IHP was calculated by first setting up an ACS at the atlantooccipital joint in the same orientation as the PNS ACS. C2 marker position was calculated relative to this atlantooccipital ACS. The arctangent of the ratio between the *y* and *x* axis coordinates was calculated to get the angle between the *x* axis of the atlantooccipital ACS (parallel to basioccipital plane) and a line running from the atlantooccipital ACS origin to the C2 marker. This quantifies instantaneous head posture in a manner comparable to our static measurement. Most instantaneous head-neck movement was concentrated at the atlantooccipital joint in both individuals based on X-ray video observation, consistent with other quadrupedal mammals [47].

### (g) Statistical analyses

All statistical analyses were conducted in R (V4.1.1, [48]). If animals are capable of adjusting head posture to modulate static hyoid position for optimal swallowing performance, two assumptions must be met: in the absence of active muscle control, hyoid position and hyolingual muscle lengths must be significantly correlated with IHP, and static hyoid position and hyolingual muscle lengths must be significantly different between different IHPs. Here we used two different statistical workflows to test each of these assumptions.

To test the hypothesis that head posture significantly impacts static hyoid position and suprahyoid muscle lengths across gapes, we employed a linear mixed effect model from the package lme4 [49]. Either hyoid *xyz* coordinates or muscle strain was designated as response variables. Head flexion angle, gape angle, and their interaction were fixed effect variables and individual animals were random effect variables. The response and fixed effect variables are both treated as continuous variables. For a given fixed effect variable, we assessed regression coefficient significance with a likelihood-ratio test between the full model with the variable included and a model without it. If the hypothesis is supported, we expected to see a significant likelihood-ratio test result between the full model and a model with head flexion angle removed. A significant interaction effect between head flexion angle and gape angle would indicate that the effect of head posture on static hyoid position and suprahyoid muscle length differs across different gapes.

While head posture and/or gape may be significantly correlated with static hyoid position, it does not predicate that static hyoid position would be significantly different between head postures and/or gapes. We conducted two sets of Friedman tests to evaluate this latter hypothesis. One set was conducted within subgroups of gape and the other within subgroups of head posture. Within subgroups of gape, we tested whether hyoid *xyz* coordinates and suprahyoid muscle strain are significantly different across different head postures. Within subgroups of head posture, we tested whether hyoid *xyz* coordinates and suprahyoid muscle strain are significantly different with different gape. Unlike the linear mixed effect models, the Friedman tests treated gape and head flexion both as discrete grouping variables, but the hyoid position and muscle strain remain as continuous variables. Sub-setting the full dataset for Friedman tests helped us better interpret the statistical results and address potential interaction effects between posture and gape (e.g. hyoid position may be statistically different with posture at one gape but not another), although it undermined the statistical power and increased Type II error likelihood. If a significant group difference was found, we conducted post hoc multiple pairwise comparisons with Bonferroni correction to examine which two groups were contributing to the observed significance.

We used degree of stereotypy [50] to test the hypothesis that hyoid kinematics during swallowing may significantly vary across different head postures. We calculated coefficient of variation (CV*) for maximum hyoid excursion distance in AP, SI and diagonal direction. CV* was calculated as (1 + 1/4*n*) × CV [51], where *n* is number of swallows. CV* corrects for the bias of estimating variance at small sample size compared to standard CV [51]. Maximum hyoid AP and SI excursions were calculated as the difference between hyoid position at swallowing cycle onset and maximum hyoid *x* and *y* position, respectively. Maximum hyoid diagonal excursion was defined as $\sqrt{{\left(\max\mathrm{AP}\;\mathrm{excursion}\right)}^{2}+{\left(\max\mathrm{SI}\;\mathrm{excursion}\right)}^{2}\;}$. We also calculated CV* for duration of hyoid protraction, elevation, retraction and depression during swallow cycles. Hyoid protraction and elevation durations were defined as the time between cycle onset and the time when hyoid reaches maximum *x* and *y* position, respectively. Hyoid retraction duration was defined as the time between maximum hyoid *x* position and the time when hyoid reaches its most posterior position before moving anteriorly again. Hyoid depression duration was defined as the time between maximum hyoid *y* position and the time when hyoid reaches its most inferior position before moving superiorly again.

## 3. Results

### (a) Change in static hyoid position across posture-gape combinations

Change in head flexion angle was the main driver of change in static hyoid position in *Didelphis* (figure 2*a*), with the largest changes along the AP axis (table 1; figure 2*b*). At a given gape, the hyoid was at the same level as the paroccipital process behind the mandible at extended head posture. Its position became increasingly rostral with decreasing head flexion angle (figure 2). The shift in static hyoid AP position across posture-gape change is comparable to, if not larger than *in vivo* hyoid protraction magnitude during swallow cycles (figure 2*a*). We found a significant negative correlation between hyoid AP position and head flexion angle (*p* *<* 0.05) and a significant negative interaction effect between head flexion angle and gape on hyoid position (*p* *<* 0.05; electronic supplementary material, figure S2). The negative effect of head flexion angle on hyoid AP position was accentuated with larger gape. At small gape, mean hyoid AP position shifted forward by 14.57 ± 5.03 mm from extension to flexion, and by 21.30 ± 6.13 mm at large gape. Across all gapes, hyoid AP position differed significantly between extended and flexed posture (*p* *<* 0.05).

**Table 1.** Mean hyoid position (mm) and suprahyoid muscle lengths (△*l/l_i_*) at different posture-gape combinations. (Standard deviations are listed in parentheses.)

gape	posture	mean AP position	mean SI position	mean ML position	mean geniohyoid strain	mean stylohyoid strain
small	extended	−34.89 (3.88)	−16.38 (1.26)	−0.26 (1.21)	0.25 (0.07)	−0.41 (0.07)
	neutral	−25.16 (3.99)	−20.37 (2.29)	2.41 (2.62)	0.04 (0.05)	0.02 (0.06)
	flexed	−20.32 (3.19)	−18.57 (1.74)	2.61 (2.23)	−0.04 (0.03)	0.10 (0.07)
medium	extended	−39.90 (4.57)	−18.02 (2.12)	0.42 (0.84)	0.25 (0.07)	−0.40 (0.04)
	neutral	−26.36 (3.90)	−21.24 (1.77)	2.07 (1.73)	0.00 (0.00)	0.00 (0.00)
	flexed	−20.24 (3.80)	−18.01 (2.13)	1.68 (1.78)	−0.06 (0.02)	0.10 (0.07)
large	extended	−43.08 (4.99)	−18.85 (2.15)	0.50 (1.34)	0.23 (0.07)	−0.36 (0.05)
	neutral	−28.76 (3.67)	−22.02 (2.11)	1.61 (1.77)	−0.02 (0.02)	−0.02 (0.04)
	flexed	−21.78 (3.55)	−19.15 (1.71)	1.70 (1.81)	−0.09 (0.03)	0.07 (0.07)

At extended and neutral posture, hyoid AP position also differed significantly between small and large gapes (*p* *<* 0.05). Mean hyoid AP position decreased by 8.18 ± 6.33 mm and 3.60 ± 5.42 mm, respectively, from small to large gapes at extended and neutral posture.

Static hyoid SI position changed much less with head posture than static hyoid AP position and *in vivo* hyoid SI excursion (table 1; figure 2*a,c*). Linear mixed effect models found no correlation between either head flexion or gape angles and static hyoid SI position. At small gape, Friedman tests found a significant difference between hyoid SI position at neutral and extended head postures (*p* *<* 0.05), and a significant difference between neutral and flexed head posture at large gapes (*p* *<* 0.05). While there was a significant difference between hyoid SI position at different head postures at medium gape, post hoc comparisons were insignificant after Bonferroni correction. At extended and neutral posture, there was also a significant difference between hyoid SI position at small versus large gapes (*p* *<* 0.05).

Mean hyoid ML position also changed little (<4 mm) from head extension to flexion across all gapes (table 1). We found no significant difference in hyoid ML positions across any gape-posture combinations, although we recovered a significantly negative correlation between hyoid ML position and head posture (*p* *<* 0.05) with a shallow regression slope (<−0.05). The hyoid seemed to deviate slightly rightwards with increasing head flexion, probably because the animals were lying on their right side during data collection on neutral and flexed head posture.

**Figure 3.**
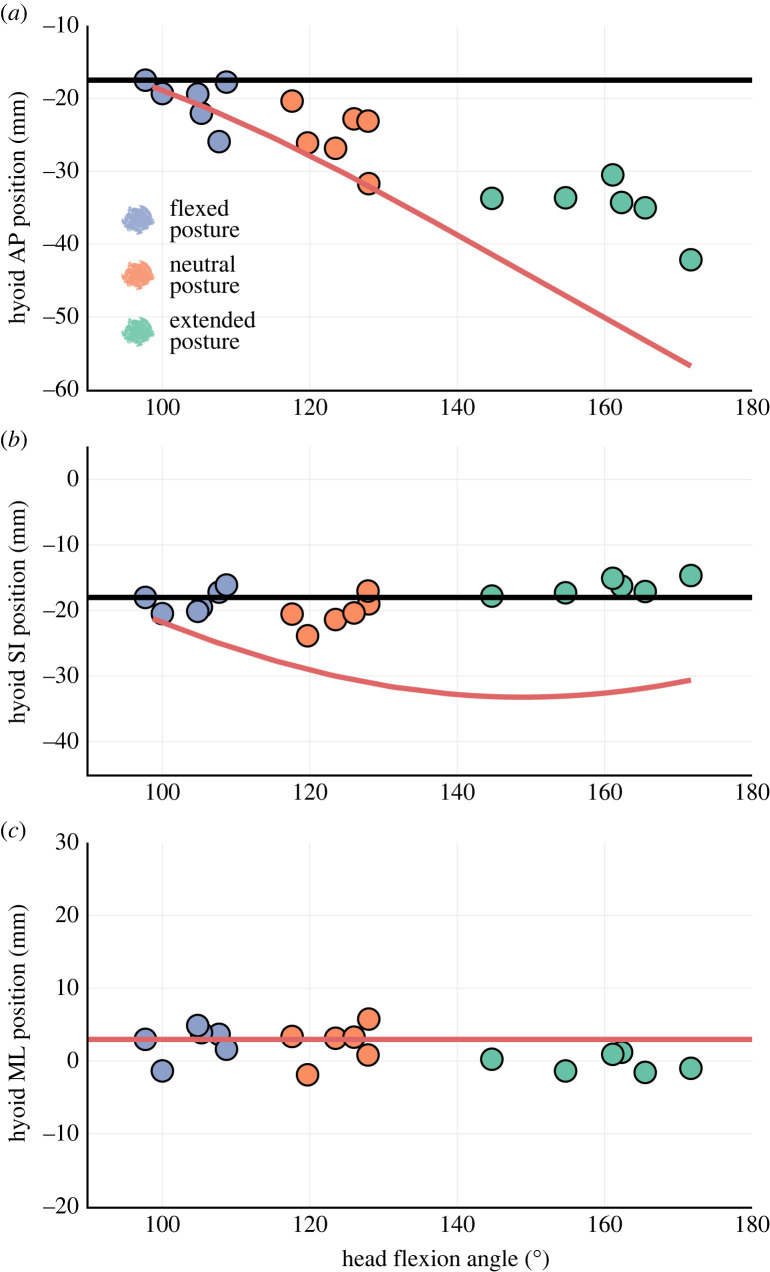
Coupling between static hyoid position and head posture varies across anatomical axes. Observed hyoid position across head posture change at small gape is plotted along AP (*a*), SI (*b*) and ML (*c*) axes against theoretical expectations of hyoid position. Data for medium and large gape are shown in the electronic supplementary material, figure S4. Horizontal lines (black in (*a*) and (*b*), red in (*c*)) show theoretical hyoid position if it is perfectly coupled to the cranium, while red curves in (*a*) and (*b*) represents theoretical hyoid position if it is perfectly coupled to the vertebral column.

The small magnitudes of hyoid position change along SI and ML axes across different posture-gape combinations suggest the effect of head posture and gape on hyoid SI and ML position is unlikely to be biologically relevant. This is confirmed by our theoretical modelling results (figure 3). Across head postures at given gape, hyoid SI and ML positions closely followed the trajectories expected under a perfect coupling relationship between hyoid and cranium (figure 3*b,c*). By contrast, hyoid AP position across different head postures deviated from both expected trajectories (figure 3*a*), suggesting that the hyoid position is neither perfectly coupled to changes in head flexion nor perfectly fixed to the cervical vertebral column. Hyoid position relative to cranium is not constant across IHP changes.

### (b) Suprahyoid muscle length change across posture-gape combinations

**Figure 4.**
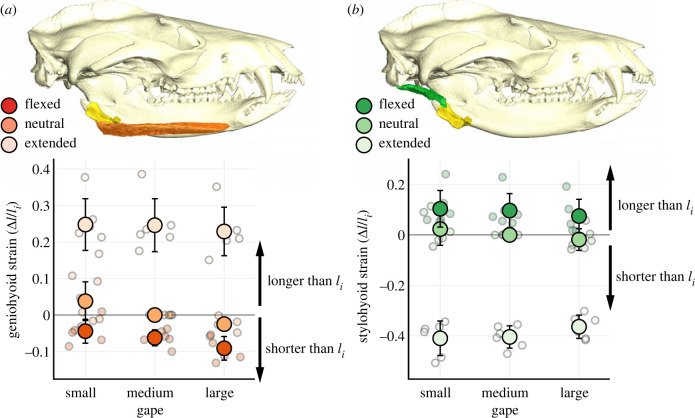
Head posture change significantly affects resting lengths of geniohyoid (*a*) and stylohyoid (*b*) muscles in *Didelphis*. Muscle strain is calculated as the difference between observed muscle length and *l_i_*, standardized over *l_i_*. diceCT visualization of genio- and stylohyoid muscles in *Didelphis* is shown in association with measurements of muscle length. Mean muscle strain is plotted over individual measurements. Error bars are standard deviations.

The lengths of both genio- and stylohyoid muscles varied significantly across different posture-gape combinations (table 1; figure 4). We found a significant positive correlation between geniohyoid length and head flexion angle (*p* *<* 0.05), as geniohyoid lengthened with increasing head flexion angle. Across all gapes, geniohyoid length was significantly shorter at flexed than extended posture (*p* *<* 0.05). At small gapes, mean geniohyoid length changed from 25 ± 7% longer to 4 ± 3% shorter than *l_i_* from head extension to flexion. At large gapes, mean geniohyoid length went from 23 ± 7% longer to 9 ± 3% shorter than *l_i_* (length at neutral head posture) from head extension to flexion.

At neutral head posture, geniohyoid length was significantly shorter at large gape than small gape (*p* *<* 0.05), with mean geniohyoid length decreased by 6 ± 5.4% from small to large gape. The effect of gape change on geniohyoid length at other head postures was minor, and gape angle was not significantly correlated with geniohyoid length (*p* = 0.059).

Both head flexion and gape angles were negatively correlated with stylohyoid length with a significant positive interaction effect (*p* *<* 0.05). Increasing gape attenuated the negative effect of head flexion angle on stylohyoid length. Across all gapes, stylohyoid length was significantly longer at flexed posture than extended posture (*p* *<* 0.05; figure 4). At small gapes, mean stylohyoid length went from 41 ± 7% shorter to 10 ± 7% longer than *l_i_* from head extension to flexion. At large gapes, mean stylohyoid length went from 36 ± 5% shorter to 7 ± 7% longer than *l_i_* from head extension to flexion.

We found significant differences in mean stylohyoid length between different gapes at a given head posture (table 1; figure 4). However, none of these differences was significant after Bonferroni correction. Moreover, post hoc visualization of interaction effects between head posture and gape suggests changes in coupling relationship between gape and stylohyoid length with different head postures (electronic supplementary material, figure S3); gape only had a marginal negative effect on resting stylohyoid length at flexed postures. Therefore, compared to the effect of head posture change, we consider any effect of gape change on stylohyoid length to be minor and not biologically meaningful.

### (c) *In vivo* head posture during swallowing and hyoid kinematics

**Figure 5.**
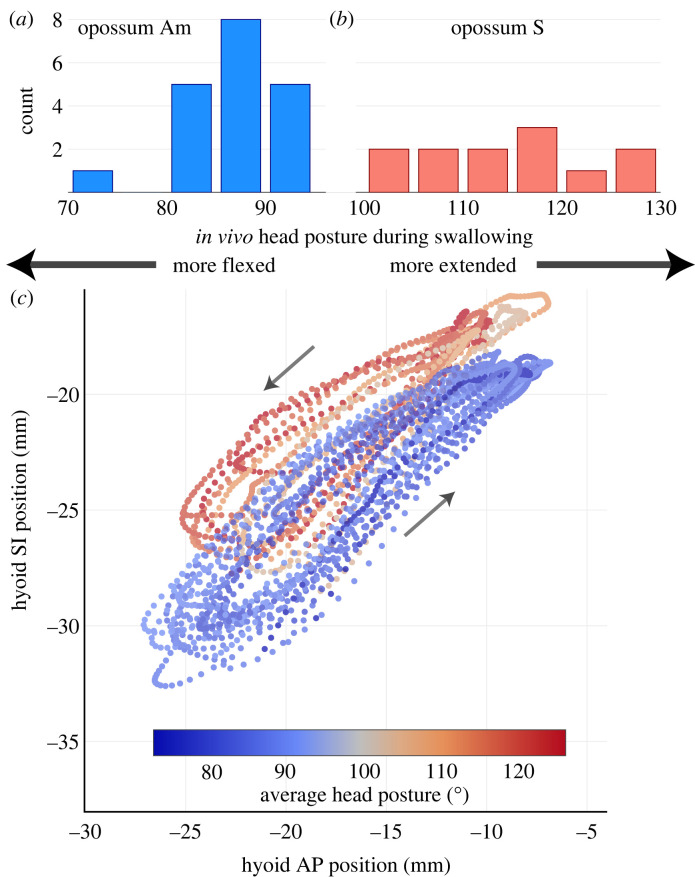
*Didelphis* adopts a neutral to flexed head posture during swallowing (*n* = 31 swallows from two individuals), with stereotyped hyoid trajectories across an observed range of swallowing head postures. (*a*) Opossum Am swallows with average head posture ranging from 70–95°. (*b*) Opossum S swallows with head posture ranging from 100–130°. (*c*) shows hyoid trajectory in right lateral view during swallowing across different head postures in both individuals. Each trajectory is coloured based on the average head posture during each swallow. Arrows indicate the direction of hyoid travel.

We observed some inter-individual variation in head posture during swallowing. While opossum Am habitually swallowed with a flexed posture (70–95° average within-cycle head flexion angle), opossum S swallowed with a posture that ranged from flexed to more neutral (100–130° average within-cycle head posture) (figures 1 and 5). However, no swallow occurred with an average within-cycle head flexion angle beyond 130° in either individual. This value is far below the range of head flexion angles in our extended posture group in the static manipulation experiments (figure 1*b*). Instantaneous head posture remained largely constant throughout a swallow. *In vivo* ranges of gape angle and mean within-cycle head flexion angle during swallowing cycles is much smaller than the static manipulations (figure 1*b*).

During swallowing, the hyoid took a counterclockwise elliptical trajectory in right lateral view (figure 5). The hyoid moved upwards and forwards initially in the swallow cycle before retracting and depressing to the starting position. There is some variation in hyoid trajectory leading up to the maximum excursion point, or during the return phase. As the hyoid moved close to its most anterior and superior position, however, individual trajectories from different swallow cycles increasingly converged upon each other (figure 5). Hyoid excursion distance and timing between different swallowing postures are similar within and between individuals (figure 5; electronic supplementary material, figures S5–S6 and table S2). On average, the hyoid protracted by 11.41 ± 2.02 mm and elevated by 10.55 ± 1.13 mm during a swallow (electronic supplementary material, table S2). Species-average CV* for hyoid excursion distance and timing ranged between 10%–20% (electronic supplementary material, table S3).

## 4. Discussion

IHP has a major effect on *Didelphis* hyoid position relative to the cranium. Across different gapes, cranial shifts in relative hyoid position with increasing head flexion are comparable to or even larger than *in vivo* hyoid protraction distance during swallowing (figure 1*a*). Our data falsify the assumptions of previous techniques to correct for IHP effect on hyoid position [15]. We demonstrate that changes in static hyoid position are not perfectly coupled to changes in head flexion, and the hyoid-cranium spatial relationship can vary extensively across changing head posture (figure 3). In support of previous studies [11,13–15], we suggest head posture should be explicitly accounted for in comparative studies for more robust inferences on inter-individual and inter-clade disparity in resting hyoid position.

Shifting hyoid–cranium spatial relationships across IHPs also impacts the lengths of two suprahyoid muscles important for hyoid movement [36,37]: geniohyoid and stylohyoid (figure 4). Consistent with previous studies [24,35], geniohyoid passively shortened and stylohyoid passively lengthened with increasing head flexion. While the biggest muscle length change occurred between extended and neutral/flexed head posture, muscle lengths also differed by 5–10% of *l_i_* between neutral and flexed posture in static conditions, which overlap with observed *in vivo* swallowing head postures (figures 4 and 5; table 1). During human swallowing, genio- and stylohyoid actively shorten by 29.2% and 21.3% on average, respectively [52]. Comparable shortening magnitudes for both muscles are reported in pigs [10] and macaques [12]. Passive strains in *Didelphis* genio- and stylohyoid from head extension to flexion rival *in vivo* length change during swallowing in other mammals [10,12,52], and the theoretical limit of active contraction by vertebrate skeletal muscle (approx. 30% [23,53]).

At the level of single muscles, IHP's effect on suprahyoid muscle resting length can impact its capacity for both passive and active force generation [54]. As suprahyoid muscle resting lengths vary with head posture, their positions on the passive and active length-tension curve will shift as well. In the case of active length-tension curves, the plateau of force generation only spans ± 10% the length for maximal isometric tension (*l*_0_). Any strain >10% will lead to a noticeable drop in force production. Moreover, head posture change has opposite effects on the lengths of different suprahyoid muscles (figure 4). It is possible that most suprahyoid muscles can operate close to *l*_0_ within a range of head posture during swallowing. Alternatively, IHP may prevent different suprahyoid muscles from simultaneously occupying *l*_0_, thus posing a trade-off between stylohyoid force generation for hyoid elevation and geniohyoid force production for hyoid protraction. New insights into the length-tension relationships of mammalian hyoid muscles [1] across different head postures are required to better evaluate if such trade-offs between individual suprahyoid muscle force production actually exists.

IHP can also reorient the force vectors of supra- and infrahyoid muscles, impacting their capacity to act synergistically or antagonistically (electronic supplementary material, figure S7) [35]. At extended posture the lines of action of supra- and infrahyoid muscles point in opposite directions [35]. For hyoid protraction and retraction, each muscle group has to generate larger force to overcome stronger resistance against the other, thereby performing more work to move the hyoid for a given distance. Head flexion repositions the lines of action between supra- and infrahyoid muscles and both muscle groups could act synergistically to depress the hyoid ventrally [35]. Changing head posture can thus affect the orientation and magnitude of resultant force produced by supra- and infrahyoid muscles.

Despite these theoretical considerations, the impact of IHP on *in vivo* hyolingual muscle function during swallowing requires further investigation. The results presented here are not informative to this question. The range of observed *in vivo* swallowing head postures is only a subset of the full range of head posture examined in static manipulations (figures 1*a*, 5), and IHP-induced variation in static hyoid position across swallowing cycles may be relatively small. Both *Didelphis* individuals consistently swallowed at a flexed-to-neutral head posture. Even the most extended *in vivo* swallowing head posture is within the ‘neutral’ range in static manipulation experiments (approx. 130°). While we observed some inter-individual variation in swallowing head posture, this is most likely owing to body size difference between individuals. Opossum Am is slightly larger than opossum S and may have needed a more flexed posture to better access the food bowl on the recording chamber floor. Within the observed head posture range, both *Didelphis* individuals exhibited similar hyoid trajectories, excursion distances and timing (figure 5). This is consistent with *in vivo* data observed in humans, where average hyoid trajectory during swallowing is similar between neutral and flexed head posture, and only differs significantly when subjects tucked their chins beyond a flexed head posture [29]. The CV* for *Didelphis* hyoid excursion timing during swallowing is also comparable with cyclic limb and jaw movement in mammals during steady-state locomotion [63] and mastication [64].

The highly stereotyped hyoid kinematics across different swallowing head postures indicates several possibilities: (i) insufficient variation in IHP was induced to meaningfully perturb hyoid kinematics; (ii) the swallowing motor programme is maintaining hyoid kinematics in the face of changing IHP; and (iii) hyoid motor control is entirely oblivious to IHP variation and its effect on hyoid muscle force production. Constraining *Didelphis* to swallow at more extended head postures than currently observed would better address possibility (i). Both possibilities (ii) and (iii) suggest a scenario in which any IHP-dependent change in hyoid muscle force production is not the major factor determining *in vivo* hyoid kinematics and swallowing performance at large. Multiple performance metrics exist for a safe swallow. These can include hyoid excursion magnitude [21,55], hyoid excursion velocity [56,57], and the precision of hyoid movement across space and time [12,18]. Force production from individual muscles is no doubt relevant for sufficient mechanical outputs to satisfy these performance metrics. However, other factors including muscle activity timing [21,58], contractile patterns [10,18,59], and sensory feedbacks from the oral and pharyngeal soft tissues [60,61] may be just as, if not more, critical for proper hyoid motor control during swallowing. Proprioceptive feedback about instantaneous head posture and/or adaptive motor learning pathways may be also important for modulating hyoid kinematics against perturbation from IHP change [65]. Integrative approaches incorporating hyoid kinematics, hyoid muscle electromyography and contractile behaviours across different head postures would further clarify the extent to which IHP is featured in swallowing motor programmes, and what aspects of hyoid muscle performance impose the strongest constraint on the range of viable head postures for safe swallowing.

## 5. Conclusion

In *Didelphis,* changes in IHP have major impacts on static hyoid position and resting lengths of suprahyoid muscles across gapes. The magnitude of passive hyoid position change owing to IHP is comparable to active hyoid excursion during swallowing in other animals [18,21,23,66]. Our results on the coupling relationship between IHP and hyoid position strengthen previous results from species with a diverse range of craniofacial and hyoid skeletal morphology [11,13–15]. We suggest this is a general feature of hyoid-skull relationships in mammals, independent of clade-specific variation in skeletal or soft-tissue anatomy. Our findings suggest the spatial relationship between hyoid and skull can be modulated via head posture change independent of hyolingual muscle actuation. The extent of head posture change on *in vivo* hyolingual muscle performance during swallowing and other complex behaviours merits further investigation.

## Data Availability

All data for static hyoid position and suprahyoid muscle length measurements are provided in the electronic supplementary material [67]. Kinematic data are available upon request.
